# The limits to population density in birds and mammals

**DOI:** 10.1111/ele.13227

**Published:** 2019-02-06

**Authors:** Philip A. Stephens, Marcus V. Vieira, Stephen G. Willis, Chris Carbone

**Affiliations:** ^1^ Conservation Ecology Group Department of Biosciences Durham University South Road Durham DH1 3LE UK; ^2^ Departamento de Ecologia Instituto de Biologia Universidade Federal do Rio de Janeiro Cp 68020 Rio de Janeiro RJ 21941‐902 Brazil; ^3^ Institute of Zoology Zoological Society of London Regent's Park London NW1 4RY UK

**Keywords:** Body size, carnivore, energetics, herbivore, macroecology, minimum viable population size, population density, scaling

## Abstract

We address two fundamental ecological questions: what are the limits to animal population density and what determines those limits? We develop simple alternative models to predict population limits in relation to body mass. A model assuming that within‐species area use increases with the square of daily travel distance broadly predicts the scaling of empirical extremes of minimum density across birds and mammals. Consistent with model predictions, the estimated density range for a given mass, ‘population scope’, is greater for birds than for mammals. However, unlike mammals and carnivorous birds, expected broad relationships between body mass and density extremes are not supported by data on herbivorous and omnivorous birds. Our results suggest that simple constraints on mobility and energy use/supply are major determinants of the scaling of density limits, but further understanding of interactions between dietary constraints and density limits are needed to predict future wildlife population responses to anthropogenic threats.

## Introduction

Understanding patterns in animal abundance is fundamental to our understanding of ecosystems and has long been a central focus of ecology (Mohr 1940; Andrewartha & Birch 1954; Damuth 1981; Peters & Raelson 1984). Previously, much attention has been paid to the relationship between body size and the average density of populations (Damuth 1981; Peters & Raelson 1984), with relatively little consideration of constraints on variation around the mean. However, understanding abundance variation and its limits provides insights into species’ tolerances to variation in resource supply (Carbone *et al*. 2011) and intraspecific variation in sociality (Smith *et al*. 1987); it also has implications for geographical distributions (Brown & Maurer 1987; Marquet & Taper 1998), trophic interactions and ecosystem function (Carbone & Gittleman 2002; Savage *et al*. 2003; Knight *et al*. 2005; Hatton *et al*. 2015), population responses to environmental change (Sutherland 1996; Bayliss & Choquenot 2002; Carbone *et al*. 2011) and conservation management (Silva & Downing 1994; Traill *et al*. 2010; Flather *et al*. 2011).

Predicting lower population limits, or minimum population densities, may provide a greater understanding of the processes influencing population viability (Silva & Downing 1994). As more populations are driven towards their lower limits, understanding where those limits lie could also aid with problems of prioritisation. Understanding how rarity varies among trophic guilds can help us to understand functional rarity (Violle *et al*. 2017) and identify which functional groups are likely to be most rare. At the other end of the scale, data on maximum density have a bearing on global trends in energy use across organisms (Enquist *et al*. 1998; Belgrano *et al*. 2002), rates of interactions between individuals (Hutchinson & Waser 2007), sociality (Macdonald 1983) and territoriality (Jetz *et al*. 2004).

A small number of studies have considered the limits to population densities, either implicitly or explicitly. In plants, maximum population density scales with individual body mass to the –3/4 power, as does average density in other organisms (Enquist *et al*. 1998). Silva & Downing (1994) examined minimum densities of mammals and found these scaled with body size to the −3/4 but at about 10% of average densities; however, they stressed the lack of a theoretical basis for predicting these findings. Silva *et al*. (1997) considered how density ranges varied with body mass, speculating that higher mobility allowed birds to achieve lower densities than similarly sized mammals. Despite this, it remains the case that, arguably, ‘we have only the haziest notion’ of what constrains lower population densities (Lawton 1990).

Here, our purpose is threefold. First, we develop simple theoretical arguments based on mobility and energy supply/demand to predict limits to the population densities of two well‐studied groups of terrestrial vertebrates: birds and mammals. We assume maximum densities are limited by resource or energy availability (Lawton 1990), while minimum densities depend on foraging and behavioural interactions, constrained by movement rates. Our simple models allow qualitative predictions regarding differences in the limits to bird and mammal population densities, both in terms of magnitude and scaling with body mass. Second, we collate extensive data sets on both bird and mammal population densities and use these to examine our predictions regarding the limits to density, and to compare them with empirical limits. The locations of upper and lower quantiles identify ‘population scope’, the range of different population densities at which most species of a given body mass are found. Third, we use our data to determine whether foraging guilds differ in relation to the predicted limits to population density.

## Methods

### Predicting minimum population density

A population should be spatially contiguous (Wells & Richmond 1995), meaning that its minimum density is the reciprocal of the maximum possible area utilised by an individual. The home range area, *A,* used by an individual is likely to relate to the distance that it travels each day, *D*: an individual of a given species that uses a larger home range will travel further each day in doing so (Garland 1983). Here, *A* is home range (in km^2^), while *D* is the length of an animal's daily travel path (in km), equivalent to the daily movement distance of Garland (1983), or the day range of Carbone *et al*. (2005). Obviously, the time of year at which daily travel is measured, and time frame over which area use is assessed, are likely to affect the apparent relationship between the two parameters in animals. We assume that these are usually assessed and reported at ecologically meaningful scales for animals of given size (Fieberg & Börger 2012) and that the scaling of time dependence (Swihart *et al*. 1988) is subsumed within the body mass scaling of both *A* and *D*. We contend that, by understanding the way that *A* increases as *D* increases, we can estimate an upper limit to *D* (*D*_max_) and thereby define an upper limit to *A* (*A*_max_). By extension, therefore, we can define a lower limit to density (*N*_min_).

Within species, it is possible to conceive of different arguments to explain how *A* will increase with increasing *D*. Previous studies (Pennycuick 1979; Garland 1983; Carbone *et al*. 2005) have tended to consider the situation faced by a forager in a patchy environment with increasingly sparse resources. If the forager has a good knowledge of where the patches are located, theory suggests that the area required to encompass a given number of patches will increase as the square of the distance between those patches (i.e. for a given species, *A* ∝ *D* ^2^; see Supporting Information part A). Because a key element of this model is that the forager has a good knowledge of where resource patches are located, we term this the ‘targeted‐search’ model. An alternative argument is that, as resources become more sparsely distributed, a forager must maintain its intensity of searching but increase the area over which it searches for food. If a proxy for intensity of use is the number of kilometres the animal travels for each square kilometre of habitat, it follows that each new square kilometre of habitat will demand a linear increase in the distance covered; that is *A* ∝ *D*. Various conjectures can lead to this finding (see Supporting Information part A) but, because it can arise from the assumption of a systematic approach to searching, we term this the ‘systematic‐search’ model for minimum density.

Both the targeted‐search and the systematic‐search models rest on the idea that, for a given species, *A* ∝ *D* ^*x*^; the only difference is that *x *=* *2 or *x *=* *1 respectively. Given these models, we can write that, within a species:

(1) $$A=c_{M}D^{x}$$

where *c*_*M*_ is the scaling constant of the relationship. A key assumption is that this is constant for a species but can vary across species with body mass (as denoted by the subscript *M*). By extension, *A*_max_ = *c*_*M*_ *D*_max_^*x*^ and, given that the minimum density is the reciprocal of the maximum area per individual,

(2) $$N_{\min}=\frac{1}{c_{M}}{D_{\max}}^{-x}$$

Given our assumption that *c*_*M*_ varies only with body mass between species, we can estimate its value for a species by rearranging eqn (1) and evaluating that for the typical home range, A*, and daily travel distance, D*, for a species of given size. In particular,

(3) $$c_{M}=\frac{A}{D^{x}}=\frac{A^{*}}{{\left(D^{*}\right)}^{x}}$$

Because typical home range and day range are known to vary with body mass as A* = *aM* ^*α*^ and D* = *dM* ^*δ*^, respectively, we can infer from eqn (3) the body mass scaling of *c*_*M*_:

(4) $$c_{M}=\frac{aM^{\alpha }}{{\left(dM^{\delta }\right)}^{x}}=\frac{a}{d^{x}}M^{\alpha -x\delta }$$

We can use this relationship in eqn (2), together with the scaling of *D*_max_, in order to estimate *N*_min_. The scaling of *D*_max_ can be defined as that of the maximum daily travel distance. Since we are interested in the limit to this parameter, we can substitute for $D_{\max}$ the normal travel speed of the animal, *S* (in km d^−1^). *S* also scales with body mass, as *S* = *sM* ^*σ*^, and multiplying this by 1 day converts it to the distance travelled by an animal that moves continuously at its normal speed. Importantly, *S* is not the same as *D*, because *D* depends on what fraction of the day a forager spends active. Thus, *S* does not have a simple relationship with *D*. By combining the scaling of *S* with (2) and substituting (4) for *c*_*M*_, we can write:

$$N_{\min}=\frac{d^{x}}{a}M^{x\delta -\alpha }{\left(sM^{\sigma }\right)}^{-x},\text{so}$$

(5) $$N_{\min}=\frac{d^{x}}{as^{x}}M^{x\delta -\alpha -x\sigma }$$

We now have a general expression for the expected scaling of minimum density. For the systematic‐search model, where *x *=* *1,

(6) $$N_{\min}=\frac{d}{\mathrm{as}}M^{\delta -\alpha -\sigma }$$

For the targeted‐search model, where *x *=* *2,

(7) $$N_{\min}=\frac{d^{2}}{as^{2}}M^{2\delta -\alpha -2\sigma }$$

To evaluate the predictions of these models, we used published data sets on the allometric scaling of their key components (Supporting Information part B). These included normal travel speed for birds (Alerstam *et al*. 2007) and mammals (von Buddenbrock 1934), and home range area for both birds and mammals (Tamburello *et al*. 2015). For mammals, a published data set is also available for daily travel distance (Carbone *et al*. 2005). For birds, however, it was necessary to infer daily travel distance by combining information on travel speed (Alerstam *et al*. 2007) with a data set on daily duration of activity (Table S1). Uncertainty in the predicted scaling of the lower limit to population density arises from uncertainty in the scaling relationships of all of the underlying parameters, which we determined using 2000 bootstrapped resamples (see Supporting Information part C). Analyses were conducted on log scales using log to the base 10 throughout.

### Predicting maximum population density

We developed a model based on population energy use and maximum global energy production. Our logic is that potential population densities are likely to be highest in biomes within which net primary productivity (NPP) is highest (Pettorelli *et al*. 2009; Hatton *et al*. 2015). In terrestrial areas, NPP is highest in tropical forests, where it can reach averages in the order of 37.8 MJ m^−2^ y^−1^ (Pidwirny 2006). We assume that most herbivores focus largely on either above‐ground or below‐ground vegetation. The larger part of this production is above‐ground NPP (ANPP), which represents *c*. 63% of total NPP (Scurlock & Olson 2013). We assume, therefore, that one maximum limit to herbivore density will occur where a single species of herbivore consumes all ANPP annually in a highly productive biome. We further assume that this highly varied intake, including substantial components of low digestibility, will be assimilated with a relatively low efficiency, in the order of 15% (Chapin *et al*. 2011; Gates *et al*. 2013). The density of individuals supported by this energy will depend on the field metabolic rate (FMR) of the species, itself related to body mass as FMR = *fM* ^*φ*^. If ANPP and FMR are both expressed in kJ d^−1^, the maximum density of an herbivorous species, in the absence of competition, will therefore be given by

(8) $$N_{\max}=\frac{0.15\mathrm{ANPP}}{fM^{\phi }}$$

To evaluate this, together with uncertainty in the estimation of FMR, we compiled and bootstrapped FMR data from Nagy *et al*. (1999), Anderson & Jetz (2005) and Speakman & Król (2010) (Fig. S5) using the same method described in Supporting Information part C. For each bootstrap replicate, density was computed using eqn (6), and confidence intervals were taken as the bounds of the inner 95% of predictions of the upper limit to density, across all 2000 replicates.

### Collating data on population densities of birds and mammals

To test our model predictions, we compiled population density data from peer‐reviewed and grey literature (e.g. IUCN reports; Robinson & Redford 1986; Damuth 1987; Silva & Downing 1994; Pearce *et al*. 2013), as well as from compendia that provide such details for large numbers of species (e.g. Cramp *et al*. 1994; Fry *et al*. 2013; Marchant *et al*. 2013; Rodewald 2015; del Hoyo *et al*. 2017). Further bird density data came from a range of regional guides. Only sources that presented density estimates for a population were considered, disregarding information on mean densities across populations, relative abundances or population size estimates without information on area sampled. Density data were matched with body mass estimates (Damuth 1987; Lislevand *et al*. 2007; Dunning 2008; Jones *et al*. 2009) and dietary classifications (Jones *et al*. 2009; Myers *et al*. 2016) for each species. Further information on the compilation of density, body mass and dietary data is given in Supporting Information part C.

### Analysis of empirical data on population densities

To estimate the upper and lower bounds of population density–body mass relationships, we used quantile regression to find the scaling of the quantiles describing the inner 95% of the population density data (i.e. the 0.025 and 0.975 quantiles; see Supporting Information part C). We evaluated population scope as the interval between the fitted 2.5 and 97.5% quantiles of population density for animals of log body mass = 0 (corresponding to 1 kg). Uncertainty around this interval was determined using bootstrapping, as above. All data analysis and model fitting were conducted using R version 3.4.4 (R Core Team 2018).

## Results

### Predictions of the limits to population density

**Figure 1:**
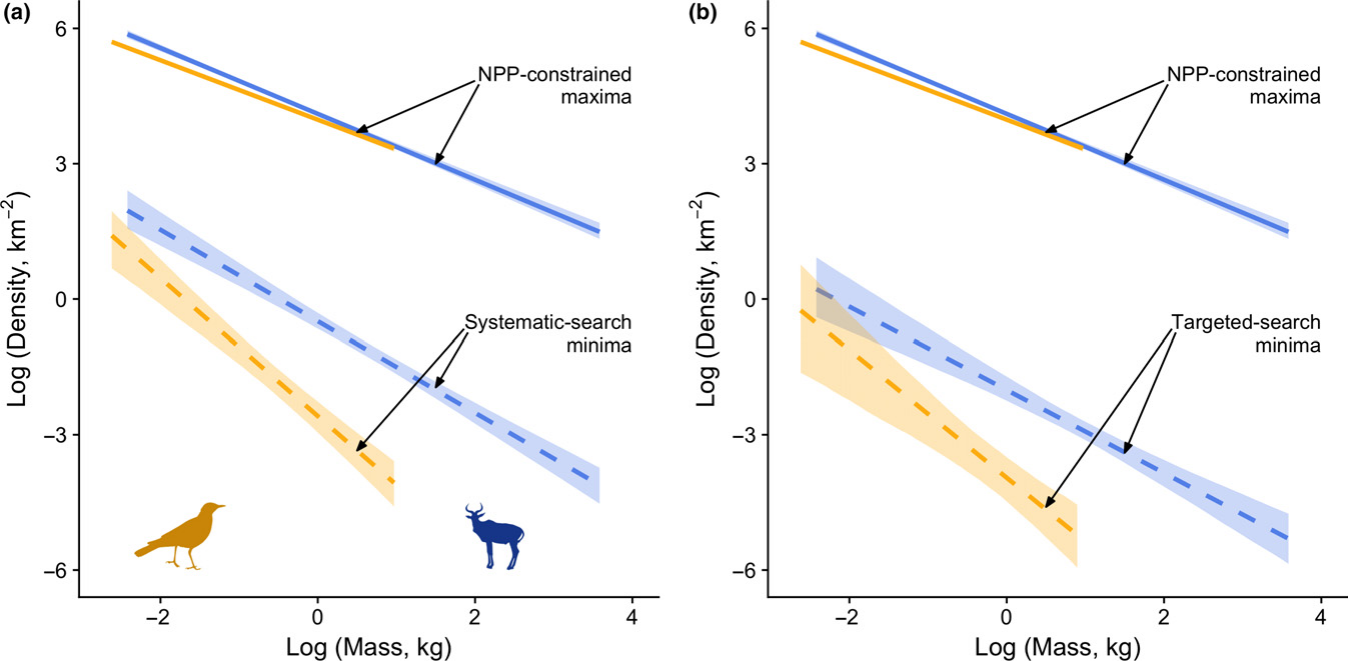
Predicted limits to the population densities of birds (gold) and mammals (blue). Upper limits (solid lines) are predicted based on energy availability and use. Lower limits (broken lines) are predicted based on mobility, assuming the systematic‐search (a) or targeted‐search (b) model for minimum density (see main text for further details). Polygons indicate bootstrapped 95% confidence intervals.

Slopes of the predicted minimum density are similar for the systematic‐search (birds, *N*_min_ ∝ *M* ^−1.53^, mammals, *N*_min_ ∝ *M* ^−1.01^) and targeted‐search (birds, *N*_min_ ∝ *M* ^−1.42^, mammals, *N*_min_ ∝ *M* ^−0.92^) models (Fig. 1, Table 1). By contrast, the two models predict very different intercepts of the *N*_min_ – body mass relationship. Under the systematic‐search model, the low bound of density is much higher than the targeted‐search model (Fig. 1). Regardless of the interpretation of minimum density, birds are predicted to be able to tolerate lower densities than mammals of the same body mass, and that boundary is predicted to scale more steeply with increasing mass in birds than in mammals.

**Table 1:** Parameters for predicted limits and fitted quantiles of population density. All fits are of the form log(*y*) = β_0_ + β_1_ log(*x*)

Description	Taxon	Guild	Intercept	Slope
Predicted upper limit based on energy supply and demand	Birds	All	3.93 ± 0.03	−0.68 ± 0.02
	Mammals	All	4.06 ± 0.03	−0.78 ± 0.02
Fitted upper (0.975) quantile	Birds	All	2.29 ± 0.06	−0.43 ± 0.04
		Herbivores	2.56 ± 0.18	−0.43 ± 0.14
		Omnivores	2.27 ± 0.09	−0.55 ± 0.07
		Carnivores	1.77 ± 0.11	−0.66 ± 0.07
	Mammals	All	2.92 ± 0.06	−0.74 ± 0.03
		Herbivores	3.23 ± 0.07	−0.80 ± 0.03
		Omnivores	2.65 ± 0.07	−0.82 ± 0.08
		Carnivores	1.91 ± 0.05	−1.02 ± 0.03
Predicted lower limit based on the systematic‐search model (*A* ∝ *D*)	Birds	All	−2.59 ± 0.17	−1.51 ± 0.14
	Mammals	All	−0.48 ± 0.09	−1.02 ± 0.07
Predicted lower limit based on the targeted‐search model (*A* ∝ *D* ^2^)	Birds	All	−3.97 ± 0.25	−1.38 ± 0.23
	Mammals	All	−2.00 ± 0.13	−0.93 ± 0.10
Fitted lower (0.025) quantile	Birds	All	−2.03 ± 0.07	−0.99 ± 0.04
		Herbivores	−0.44 ± 0.29	−0.12 ± 0.20
		Omnivores	−0.96 ± 0.11	−0.44 ± 0.07
		Carnivores	−2.37 ± 0.15	−1.13 ± 0.08
	Mammals	All	−0.94 ± 0.06	−0.79 ± 0.04
		Herbivores	−0.06 ± 0.16	−0.72 ± 0.06
		Omnivores	−0.79 ± 0.11	−0.85 ± 0.11
		Carnivores	−1.17 ± 0.14	−0.76 ± 0.08

Inevitably, our predictions of the upper limit to population density scale with the reciprocal of field metabolic rate scaling, and so are negatively related to body mass (Fig. 1). Metabolic demand is slightly higher for small‐bodied birds than for small‐bodied mammals, so the corresponding predicted maximum is slightly lower for birds; however, these limits converge for larger birds and similarly sized mammals due to a convergence of metabolic rates (Fig. 1). Based on our predictions, we would expect birds to have a higher population scope than mammals when evaluated at an intermediate size (e.g. 1 kg).

### Comparing predictions to empirical population density data

**Figure 2:**
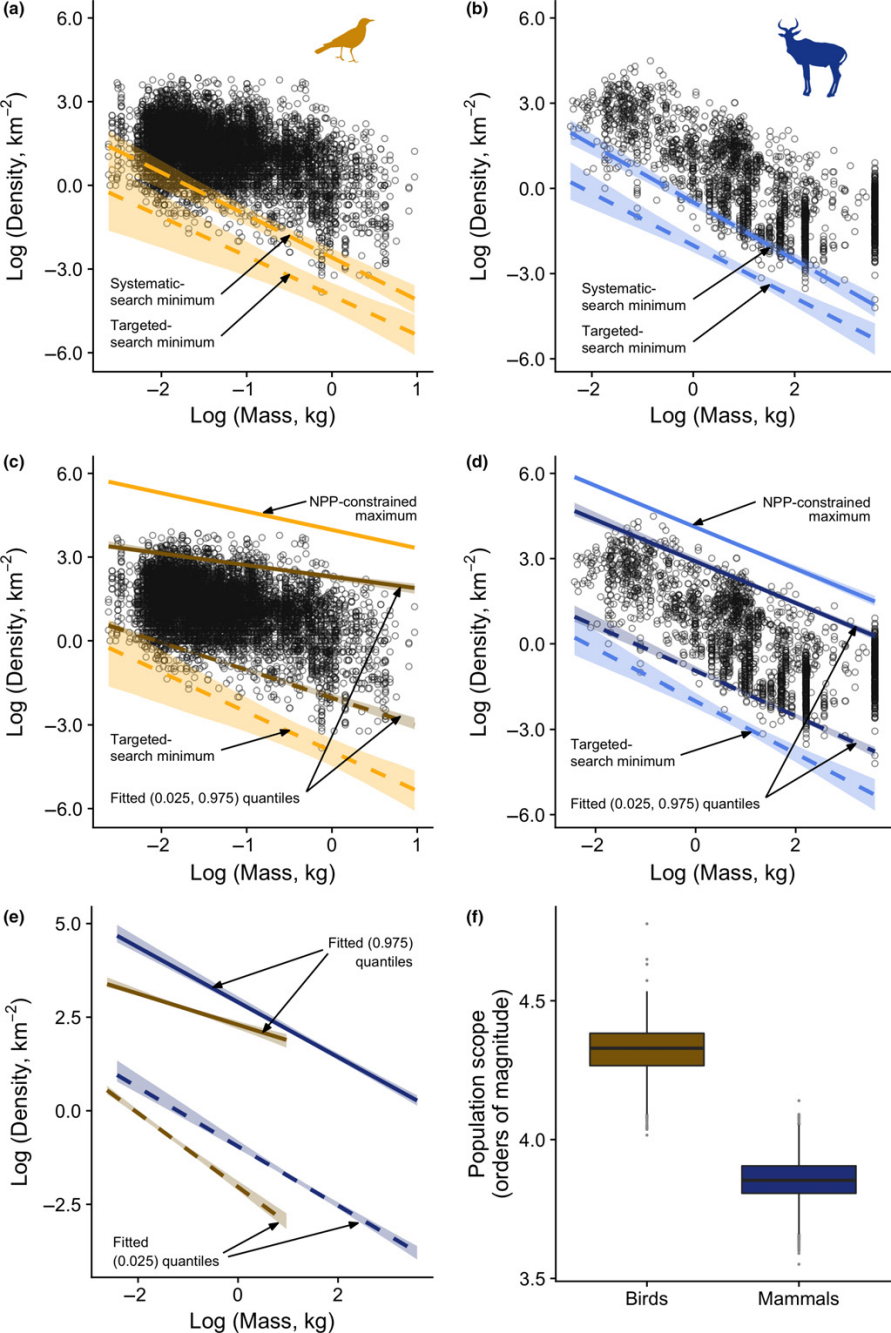
Collated data on population densities in relation to lower predicted limits for (a) birds and (b) mammals. Predicted limits are based on the systematic search (long dashes) and targeted search (dashes) models. Both upper predicted limits (solid lines) and lower predicted limits based on the targeted‐search model (dashed lines) are shown in relation to the data for (c) birds (lines in gold) and (d) mammals (lines in blue). Fitted 0.025 and 0.975 quantiles are shown for birds (dark gold) and mammals (dark blue). (e) For ease of comparison with the qualitative predictions of Fig. 1b, the fitted quantiles for birds (dark gold) and mammals (dark blue) are shown together. Polygons in panels A–E indicate bootstrapped 95% confidence intervals. (f) Empirical population scope (the range of population densities at which populations of a given body size are found) estimated for animals of 1 kg body mass. Variation in the estimates arises from the 2000 bootstrap replicates drawn from the underlying raw data. The line across each box indicates the median and the box boundaries indicate the interquartile range (IQR). Whiskers identify extreme data points that are not more than 1.5 times the IQR on both sides; the dots are more extreme outliers.

Although the two approaches to estimate the lower limit to population density agree in their major qualitative predictions, they provide rather different quantitative predictions. The systematic‐search model for minimum population density does not perform well as an absolute boundary, lying above almost 5% of empirical estimates of density (380 of 8188 bird population densities and 111 of 2286 mammal population densities lie below the boundary) (Fig. 2a and b). By contrast, the targeted‐search model appears to provide a good description of the outlying low estimates of density, with only three estimates of bird density and one estimate of mammal density lying just below the boundary (Fig. 2a and b). As these two models are likely to lie at either end of the range of possible relationships between home range and day range, and as the targeted‐search model represents a more extreme case, further inference is based on the targeted‐search model.

Using the targeted‐search predictions for minimum density, almost all field estimates of the densities of both birds (Fig. 2c) and mammals (Fig. 2d) fall within the boundaries predicted by our simple models. Populations of mammals approach the upper bound more closely than do populations of birds. Upper fitted quantiles (Fig. 2c and d) show that a failure to approach the upper boundary is even more pronounced for small birds; the upper quantile for birds is substantially lower and scales less negatively than predicted, in contrast to mammals for which it scales as predicted. At the lower limit of population density, with only the four exceptions mentioned above, populations are bounded by the targeted‐search limit. When viewed in isolation, the fitted upper and lower quantiles for birds and mammals (Fig. 2e) show the qualitative patterns predicted by our models (Fig. 1), with the exception that the upper limit to bird densities is rather lower than expected, relative to that for mammals. Nevertheless, when evaluated at a body mass of 1 kg, the emergent population scope for birds is higher than that for mammals (Fig. 2f), as we predicted.

### The influence of foraging guild

**Figure 3:**
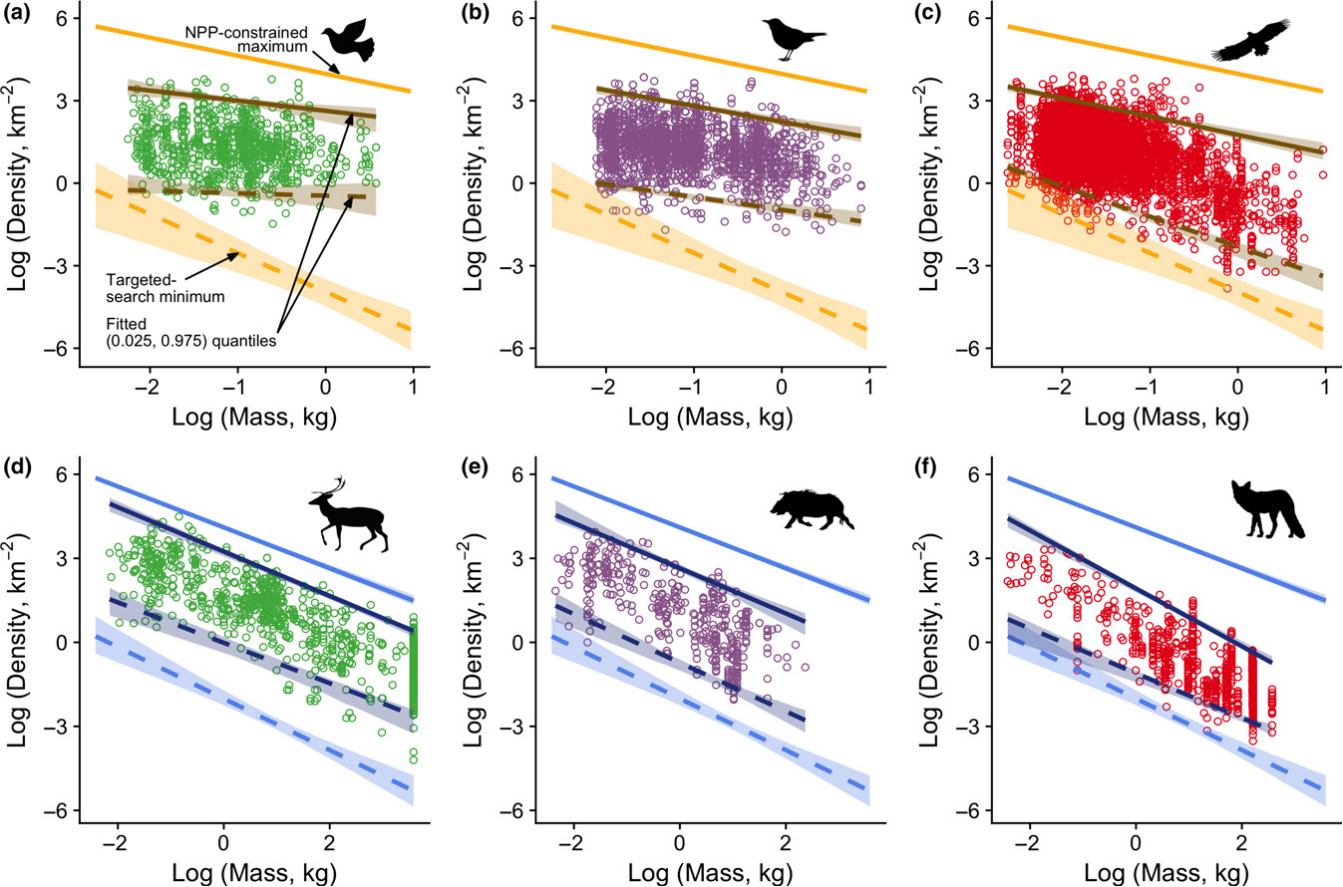
Population densities, trophic guilds and density limits for avian herbivores (a) omnivores (b) and carnivores (c), and mammalian herbivores (d), omnivores (e) and carnivores (f). In each panel, data points show the raw data on population densities. Both upper predicted limits (solid lines) and lower predicted limits based on the targeted‐search model (dashed lines) are shown in relation to the data (birds, upper panels, lines in gold; mammals, lower panels, lines in blue). Fitted 0.025 and 0.975 quantiles are shown for birds (upper panels, dark gold) and mammals (lower panels, dark blue).

The pattern of mass‐dependent variation in population density seen across birds as a whole shows striking inconsistencies among foraging guilds (Fig. 3a–c). In particular, carnivorous birds show a pattern similar to that predicted, with the upper boundary substantially lower than that based on the limit imposed by primary production, as would be expected of secondary consumers (Fig. 3c). By contrast, herbivorous birds show much less mass dependence in both the upper and lower boundaries (Fig. 3a), while omnivorous birds exhibit a pattern intermediate between the other two guilds (Fig. 3b).

Unlike foraging guilds in birds, all three of the mammalian guilds show patterns similar to those expected (Fig. 3d–f). Upper boundaries for herbivorous and omnivorous mammals show the same scaling as the predicted upper boundary; for carnivores, the scaling is slightly steeper. Fitted upper quantiles are closest to the predicted upper limit for herbivores, and progressively further away for omnivores and carnivores, as expected for these increasing trophic levels. Herbivorous and carnivorous mammals show a fitted lower quantile with a scaling slightly shallower than that predicted by the targeted‐search model, but outlying estimates nonetheless approach that constraint at relatively high body masses.

## Discussion

Studies of body size–density relationships have emphasised the scaling of mean density. Here, we go beyond a focus on mean relationships. Our data sets, among the most extensive and diverse for birds and mammals, illustrate the huge variation in population densities across species of similar size. Our quantile regression analyses suggest that density variation is related to taxon, body size and diet, while our models of absolute limits provide an important frame of reference against which to judge observed patterns in the data. We discuss these findings with reference to: the scaling of the (1) lower and (2) upper limits to population density and their relationships to our predictions; (3) the influence of foraging guild on variation in population densities; and (4) the magnitude of population scope (range in population density) and its broader implications for ecology.

### The lower limit to density

With the exception of outlying estimates for three bird and one mammal species, our simple, mobility‐based model of a targeted‐search constraint provides boundaries that describe the low range of mammal and bird population densities remarkably well. The model based on the logic that area used will increase with the square of distance travelled, consistent with previous interpretations (Pennycuick 1979; Garland 1983; Carbone *et al*. 2005), provided a more compelling description of the lower bound to population density than that based on the assumption that foragers would show a constant intensity of searching as area increased. This finding could be interpreted as evidence that most foragers exist within a patchy environment and have a reasonable knowledge of where those patches are located (akin to the ‘ideal knowledge’ of Fretwell 1972). As patches become fewer, they must increase the distance they travel between them. Our contrasting, less‐supported hypothesis would be more in line with the idea that food is highly unpredictable in space and time and, thus, as food becomes scarcer, individuals must cover more area with their usual intensity of searching. Actual search patterns may represent a combination of these processes, with our two model derivations representing different extremes. In general, for populations to show genetic and demographic (as well as spatial) contiguity (Wells & Richmond 1995), individuals must maintain rates of contact with conspecifics and, thus, range more widely than required by their normal foraging intensity. At the low end of the density spectrum, therefore, individuals might have to use space more extensively, in order to maintain rates of individual interactions sufficient to offset the behavioural processes and genetic considerations that are often invoked to explain the poor viability of low density populations (Lande 1998; Stephens & Sutherland 1999). More within‐species data on how home range varies in relation to travel distance would shed light on the assumptions of our minimum density models.

For both taxa, our models strongly suggest that large‐bodied species can tolerate lower absolute densities than can small‐bodied species. This is consistent with previous findings for mammals, and supports similar findings for birds, which have tended to be more contentious (Silva *et al*. 1997); importantly, our models suggest mechanisms to explain the influences of both body mass and taxon in these previous observations. Whether large‐bodied or small‐bodied species can tolerate lower relative densities is complex. Small‐bodied species are typically subject to more stochastic variation and might be more vulnerable to chance extinctions; however, large‐bodied species have lower intrinsic rates of increase, potentially making it hard for them to escape an extinction vortex (Lawton 1990). In practice, the sparse nature of data around the lower boundary makes it difficult to determine whether one of these processes predominates.

### The upper limits to density

Our maximum density prediction for both taxa is based on the supply of net primary production because, owing to inefficiencies of energy conversion, energy supply of other resources is likely to be lower. Our predictions suggest a similar scaling of the upper bound to density for birds and mammals but the empirical data suggest that mammals achieve higher densities than similarly sized birds, as found by Silva *et al*. (1997). As with our predicted lower limits, we did not expect populations to reach these densities. Although no estimate of bird or mammal population densities exceeded our predicted maximum density, a number of estimates for both taxa came very close. This is particularly surprising because recent evidence suggests that bird and mammal densities are likely to be maximised at moderate levels of productivity (Santini *et al*. 2018). The proximity of some estimates to the upper boundary could arise from sampling errors in the underlying data, or because: (1) local‐scale productivity is notoriously difficult to estimate and might, in many biomes, show large deviations from the mean values for maximum productivity of vegetation in those biomes; (2) we could have underestimated the assimilation efficiency of some primary consumers, despite the low digestibility of some food types that must be eaten to account for the full range of above‐ground productivity; or (3) some species might have adaptations to reduce field metabolic rate well below expectations for their body mass. Our findings highlight the challenge to estimate the relevant parameters so that it is possible to identify which of these is the commonest explanation.

### Foraging guilds and variation in population density

More insight into the processes dictating the limits to density can be gained by considering the fit of our models to the population densities of species in different foraging guilds. In this regard, Fig. 3 illustrates four phenomena that would benefit from explanation.

First, in both birds and mammals, the average gap between the upper bound fitted to empirical data and the modelled upper bound is smallest for herbivores and largest for carnivores. This is reasonably easy to explain, by invoking understanding of energy flow through ecosystems (Elton 1927; Pauly & Christensen 1995), which suggests that the maximum resources available to secondary consumers should be lower than those available to primary consumers.

Second, the fitted upper boundaries for birds do not always mirror closely the slopes of the predicted upper boundaries. In particular, the fitted upper boundary for herbivorous (and, to a lesser extent, omnivorous) birds is substantially flatter than the predicted boundary. Small birds and mammals are typically restricted to high quality diets, as a consequence of their absolute digestive capacity (Dial *et al*. 2008), and this is likely to be particularly pronounced among birds, owing to the mass restrictions of flight (Morton 1978). Consequently, it seems likely that the broad range of NPP is not as available to birds as to mammals, and not as available to small birds as to large birds. Small birds might, thus, be particularly reliant on seasonally restricted food types (such as seeds). The same observation might explain the apparent dearth of very small mammals that achieve densities approaching the predicted maximum (Fig. 3;see, also, Brown & Maurer 1987, 1989).

Third, the fitted upper boundary for carnivorous mammals is rather steeper than expected. The lack of higher density carnivores is unlikely to be a sampling artefact, given that these are the best studied species (Brooke *et al*. 2014), so the true density range should be well sampled. That these limits are not predicted by our models hints at a possible role for anthropogenic factors, which might affect larger predators more than their smaller counterparts (Ripple *et al*. 2016).

Fourth, fitted lower bounds for herbivorous and, to a lesser extent, omnivorous birds are much less mass‐dependent than predicted (Fig. 3a and b). We might conclude that our model is not supported by the data were it not for the fact that carnivorous birds achieve densities as low as predicted. This pattern of carnivores, alone, driving the negative scaling of the lower bound for birds, was also observed by Silva *et al*. (1997). Large‐bodied herbivorous birds are dominated by Galliformes (16 of the 18 species with masses in the top 20% of the range of masses in our herbivorous bird data set), which are relatively sedentary. If diet and associated digestive machinery constrain mobility in these birds, their inability to adopt the more efficient flight strategies of similarly sized carnivorous birds could also prevent them from maintaining viability when at low density.

Overall, more detailed data on the activity and ranging behaviour of birds and mammals with different dietary strategies could allow models to be derived for different foraging guilds, potentially casting light on the mechanisms responsible for differences in scaling of the observed lower boundaries to density (Fig. 3). Better data on mammalian travel speeds and, in particular, on avian activity, would be necessary to explain why carnivores in both taxa seem capable of achieving lower densities than populations of species in other guilds (Fig. 3). The dimensionality of food searching (e.g. 2D vs. 3D, Pawar *et al*. 2012) could play a role in the differences between taxa and guilds. However, within‐guild variation in the use of terrestrial, arboreal and aerial resources suggests that this is unlikely to provide a clear explanation for observed differences.

Our assessments of the influence of foraging guild provide a useful reference against which to consider densities of humans (Supporting Information part E). Human hunter‐gatherer population densities are consistent with densities of similarly sized wild mammals, even when accounting for diet (Fig. S7). At the low end of density, developing effective MVPs for highly threatened indigenous populations (Hamilton *et al*. 2014; Walker *et al*. 2016) could therefore build on findings for similarly sized wild mammals, flagging populations at obvious risk. At the high end of density, maximum modern urban densities far exceed our predicted limit to mammalian population density, exceeding the densities of even the most abundant rodent populations (Fig. S7A). This, combined with the fact that our resource use far exceeds the natural use of resources by organisms of around 60 kg, highlights the overwhelming impacts humans currently have on the planet's resources.

### Population scope and its interpretation

The scaling of upper and lower limits to density gives an indication of the range of densities at which species of a given body mass can occur – a phenomenon that we term ‘population scope’. Brown & Maurer (1987) speculated that the upper limit to density should decrease more rapidly than the lower limit with increasing body mass. This would lead to a reduction in population scope with increasing mass. Neither our models (Fig. 1) nor fitted empirical quantiles (Fig. 2e) provide support for this possibility.

Population scope is perhaps most interesting where it is clear that the empirical data on densities suggest that species are not achieving their expected scope. We have already considered several of the most striking of these instances. A less obvious departure from expectation is the dearth of low‐density populations of small‐bodied carnivorous mammals (Fig. 3f). Given that constraints arising from stochasticity seem no more likely to affect small carnivorous mammals than small mammals in other guilds, this finding is more likely to arise from sampling biases. Small carnivores are relatively understudied (Brooke *et al*. 2014), and the densities of sparse populations of these (typically) elusive species may be particularly hard to estimate. Overall, the variation in sampling effort among mammalian guilds and across body masses places significant constraints on our ability to identify genuine departures from expected patterns of density. Standardised, comprehensive and widespread approaches to estimate mammalian population densities (Steenweg *et al*. 2014) are needed to remedy these constraints.

Whatever the reasons for discrepancies between expected and reported limits to population density, it is clear that – across guilds – the population densities of mammals of a given body mass can vary by close to 4 orders of magnitude, while those of birds can vary by substantially over 4. This highlights the extreme variation in individual spatial distribution across populations, which, within each taxon, represent physiologically and metabolically similar groups of species. It also implies significant differences in rates of interaction (Hutchinson & Waser 2007), with important implications for sociality. At present, we know little about the adjustments in time and energy budgets required to maintain home ranges on such vastly different spatial scales.

## Conclusions

Our models to predict the limits to population density in birds and mammals are coarse, ignoring the idiosyncrasies of the ecologies, life histories and energetics of individual taxa. Nevertheless, they substantially improve on existing explanations for the limits to avian and mammalian population density, bounding the available empirical data and predicting scalings of the limits to density that are broadly consistent with observed minimum and maximum densities derived from quantile regression analyses. Differences in the lower bounds to bird and mammal densities, and in their relative abilities to approach the upper bounds, help to explain previous observations that birds tend to be less dense on average than similarly sized mammalian species (Silva *et al*. 1997). Overall, the models also provide insights into important differences between dietary guilds. In order to understand these patterns fully, we need greater information on ecological context in macroecological data. In particular, behavioural information on time budgets, movement, feeding ecology and competition would be invaluable to understand the implications of vast differences in spatial ecology. In turn, this will improve our ability to predict future changes in populations under changing environmental conditions.

## Authorship

PAS, CC and MVV conceived the study. All authors collated the data, and both MVV and SGW contributed to the novel data. PAS performed the analyses, with inputs from MVV, CC and SGW. All authors contributed substantially to the final text.

## Data Availability

Data available from the Figshare Repository: http://doi.org/10.6084/m9.figshare.7553999.
