# Supplementary material for: The limits to population density in birds and mammals
Source: Ecol Lett. 2019 Feb 6;22(4):654–63. doi: 10.1111/ele.13227 (PMC6850427; doi:10.1111/ele.13227)
Supplement: Supplementary file 1 [file ELE-22-654-s001.pdf]

Supplementary materials for Stephens et al.,

The limits to population density in birds and mammals

A. Two models of how foraging might affect area use

In the main text, we suggest that – for a given species – the relationship between area use, *A*, and daily travel distance, *D*, could be conceptualised in different ways. One way to think about how *A* and *D* might be related is to consider that an important determinant of increased area use is decreased food availability. In that context, the relationship between *A* and *D* depends critically on the way that food availability declines and the way that foragers search for food. To illustrate this point, we depict various scenarios of food distribution (Fig. S1). To begin with, we imagine that food might be patchily distributed in the environment, with patches randomly located at some given density. A forager might have a home range adequate to encompass some required number of patches, *m* (Fig. S1A). If we assume that the forager is efficient in its movement between patches, then the travel distance between them should approach its minimum value (D_{min}), which is given by (Marks 1948):

$$D_{min} = \frac{1}{2}\sqrt{A}\frac{m-1}{\sqrt{m}}. \quad \text{(S1)}$$

When *m* is relatively large, $(m-1)/\sqrt{m} \rightarrow \sqrt{m}$, so

$$D_{min} \rightarrow \frac{1}{2}\sqrt{A}\frac{m}{\sqrt{m}} = \frac{1}{2}\sqrt{Am}. \quad \text{(S2)}$$

This means that if *m* is held constant but *A* increases, D_{min} will increase with $\sqrt{A}$, so $A \propto D_{min}{}^2$. However, if *m* increases at the same rate as *A*, D_{min} will increase with $\sqrt{A^2}$, so $A \propto D_{min}$. The result is that, as stated above, the manner in which food availability declines has a big impact on the expected relationship between *A* and *D*. For example, if the density of food patches is reduced by half, then to maintain a constant *m*, *A* must be doubled (Fig. S1B). In that case, equation S2 tells us that D_{min} will be increased by a factor of $\sqrt{2}$; i.e., $A \propto D^2$. Because this model relies on the individual being an efficient forager with a good knowledge of where the patches are and, hence, where it needs to get to next, we call this model the "targeted-search" model. If, by contrast, each food patch becomes half as productive, the forager must visit twice as many food patches. To do that, it must cover twice the area; in that case, both the area and the number of food patches have doubled (Fig. S1C). In that case, equation S2 tells us that when *A* has doubled, D_{min} will be increased by a factor of $\sqrt{4}$; i.e., D_{min} will also have doubled, so $A \propto D$. The same finding applies to individuals that have little knowledge of where food is located and which, as a result, must search their habitat systematically. In that case, assuming a certain radius of food detection around the forager, it must pass through points that are evenly spaced in the landscape. To double the area over which it searches (assuming that it maintains

the same search strategy and the same intensity of habitat use), it must double the number of points through which it passes (Fig. S1D). Here, again, the implication is that $A \propto D$. Because this could describe the behaviour of a forager that moves systematically around its habitat, we call this model the "systematic-search" model.

Although we have developed these conceptual models in the context of foraging, in reality, the hypothesised searching behaviours could apply to any resources, even conspecifics with which to interact. The important point is that, if those resources become more spread out but the focal individual is targeted in their search, we expect that $A \propto D^2$. If, by contrast, the individual is less aware of where the resource is to be found and, hence, must search systematically, we expect that $A \propto D$.

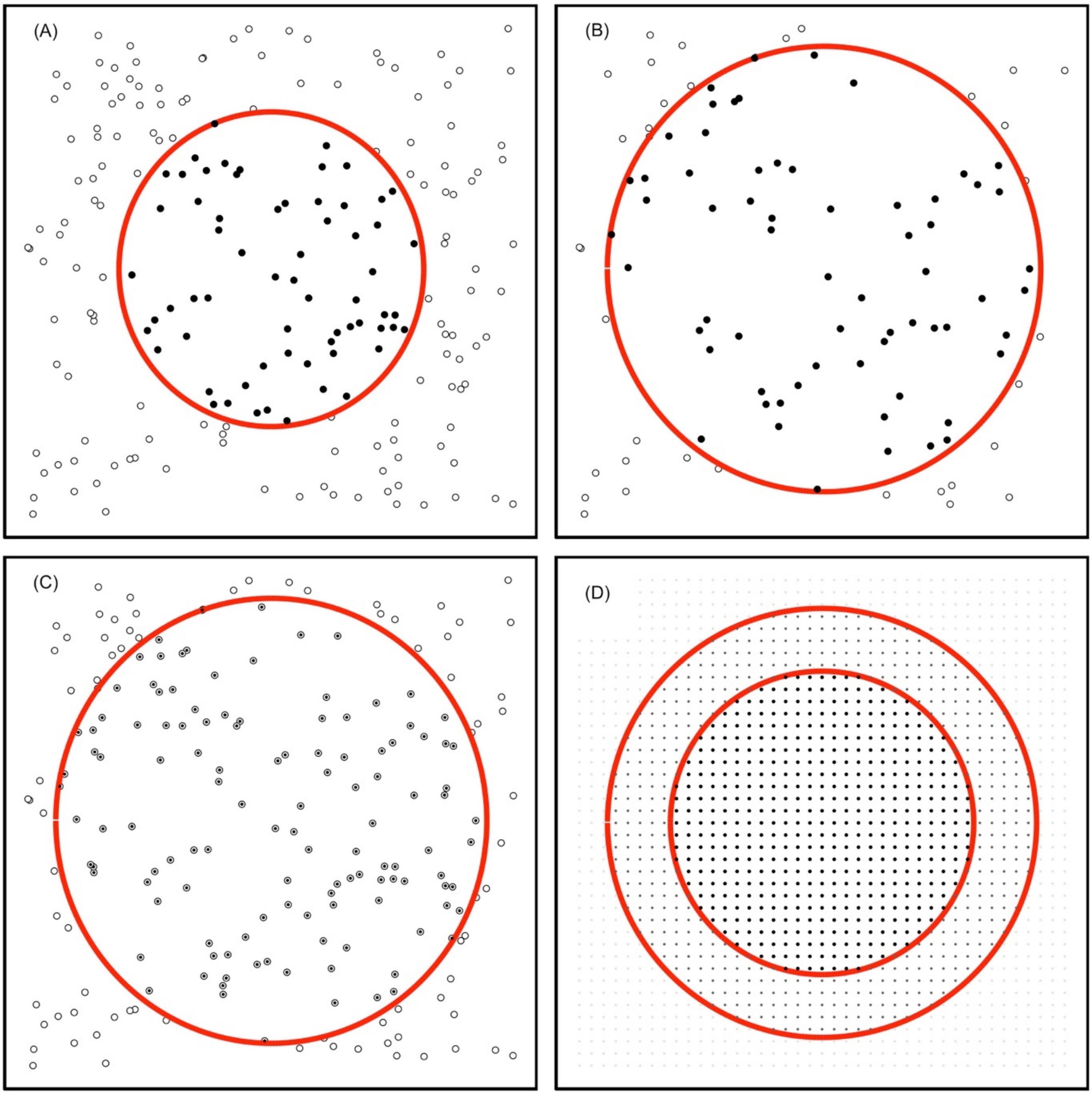

Fig. S1. Scenarios of resource distribution and home range area. (A) Resource patches (black circles) are randomly scattered in the environment and home range (red circle) is adequate to encompass *m* patches (filled circles). (B) If the density of patches in the environment declines by a half, home range must double in size to continue to encompass *m* patches. (C) If, by contrast, the density of patches remains the same but patches are halved in quality (indicated by the reduced fill of patch markers encompassed by the home range), the home range (red circle) must double in size to contain 2*m* patches. (D) If resources could be located anywhere within the environment, the searcher must cover the entire area with an intensity dictated by its radius of detection (*r*). One way to do that would be to search systematically, covering all points that are a distance of 2*r* away from any other visited point. If the frequency of finding food by this method is halved, the forager must double the area searched (from the smaller red circle to the larger red circle). If it continues to search with the same intensity as previously, it will have to visit twice as many points per unit time to find twice as many units of resource.

B. Parameters used in model development

Our models of the limits to population densities are based on the body-mass scaling of a range of underlying parameters. For both birds and mammals, our models of minimum density based on space use and mobility depended on the scaling of home range area (*A,* km^2) (Fig. S2) and travel speed (*S,* kmd^{-1}) (Fig. S3) in relation to body mass (*M,* kg). Fitted parameters (with standard errors in brackets) are:

Birds, $\log(A) = 1.22\ (\pm\ 0.13) + 1.64\ (\pm\ 0.09) \times \log(M)$

Mammals, $\log(A) = -1.03\ (\pm\ 0.07) + 1.10\ (\pm\ 0.05) \times \log(M)$

and

Birds, $\log(S) = 3.00\ (\pm\ 0.01) - 0.02\ (\pm\ 0.01) \times \log(M)$

Mammals, $\log(S) = 1.45\ (\pm\ 0.04) + 0.21\ (\pm\ 0.02) \times \log(M)$

In addition, we needed data on the scaling of day range (daily travel distances). Few data are available to suggest the scaling of day range for birds during non-migratory flight periods. To infer plausible day ranges, we determined the scaling of proportion of time active, and combined this with the scaling of travel speed. The scaling of proportion of time active was determined using published data (Table S1). This yielded the relationship between percentage of active time spent flying (*F*) by birds and body mass (*M*) (Fig. S4A):

Birds, $\log(F) = 0.93\ (\pm\ 0.11) + 0.11\ (\pm\ 0.12) \times \log(M)$

These data on flight time (and associated uncertainty) were combined with data on travel speed (and associated uncertainty) (see Fig. S3A) to derive the scaling of day range (assuming an average of 12h of potential activity). This yielded:

Birds, $\log(D) = 1.63\ (\pm\ 0.10) + 0.09\ (\pm\ 0.11) \times \log(M)$

For mammals, more data are available on day range (*D,* km) (Fig. S4B). Fitted parameters for the relationship with body mass (*M*) (with standard errors in brackets) are:

Mammals, $\log(D) = -0.07\ (\pm\ 0.04) + 0.30\ (\pm\ 0.03) \times \log(M)$

For estimating the upper bound to density, we used data on the scaling of field metabolic rate (*FMR,* kjd^{-1}) in relation to body mass (*M*). Data were obtained from Nagy *et al.* (1999), Anderson & Jetz (2005) and Speakman & Król (2010). Duplicates were removed to yield 276 estimates of avian FMRs and 228 estimates of mammalian FMRs. Where multiple data were available for a species, both mass and FMR were averaged across all records for that species. The final data set included FMR estimates

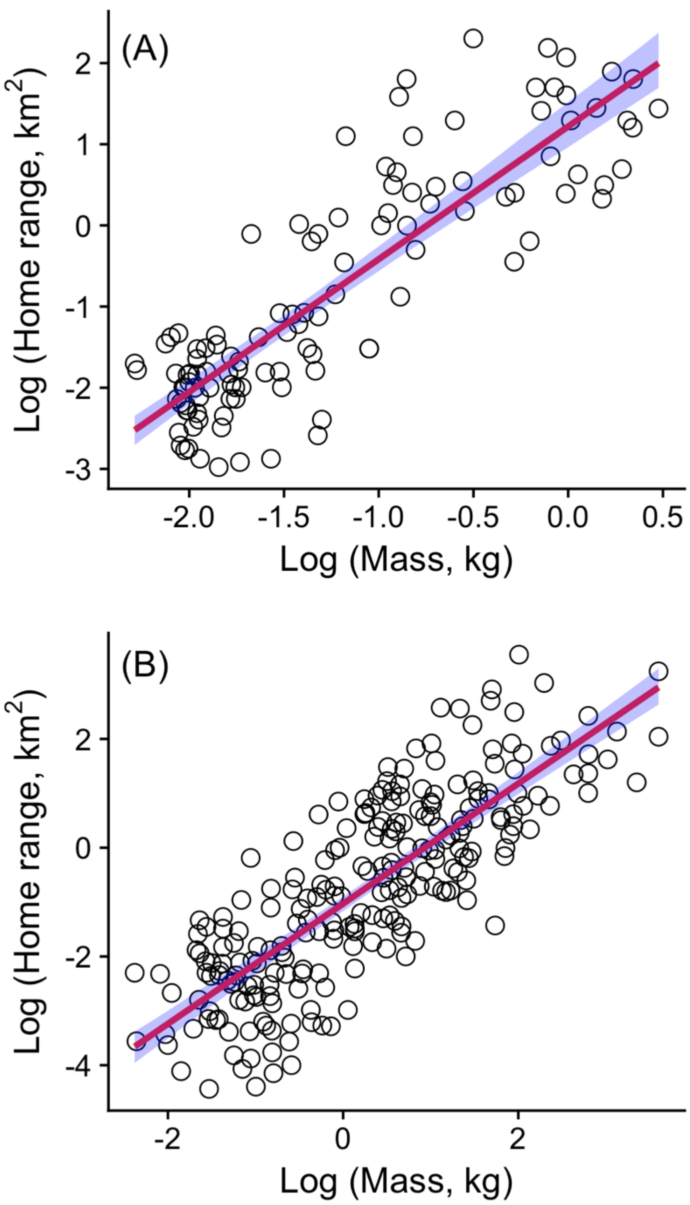

Fig. S2. Fitted ordinary least squares regressions (red lines) for home range size (Tamburello *et al.* 2015) (*A*) of birds (panel A) and mammals (panel B) in relation to body mass (*M*). Blue polygons show 95% confidence limits determined by bootstrapping (as described in the main text). For birds, data were restricted to flying species, using the designations in (Tamburello *et al.* 2015).

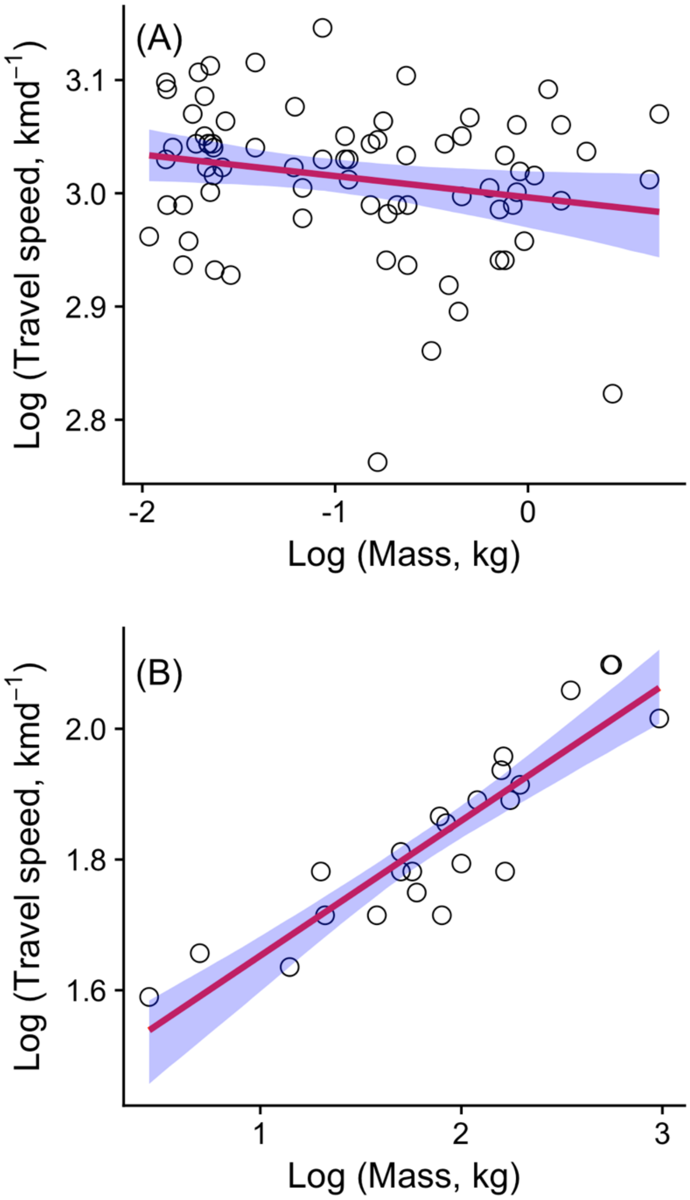

Fig. S3. Fitted ordinary least squares regressions (red lines) for travel speed (*S*) of birds (Alerstam *et al.* 2007) (panel A) and mammals (von Buddenbrock 1934) (panel B) in relation to body mass (*M*). Blue polygons show 95% confidence limits determined by bootstrapping (SI part B). We excluded travel speed data on waterbirds.

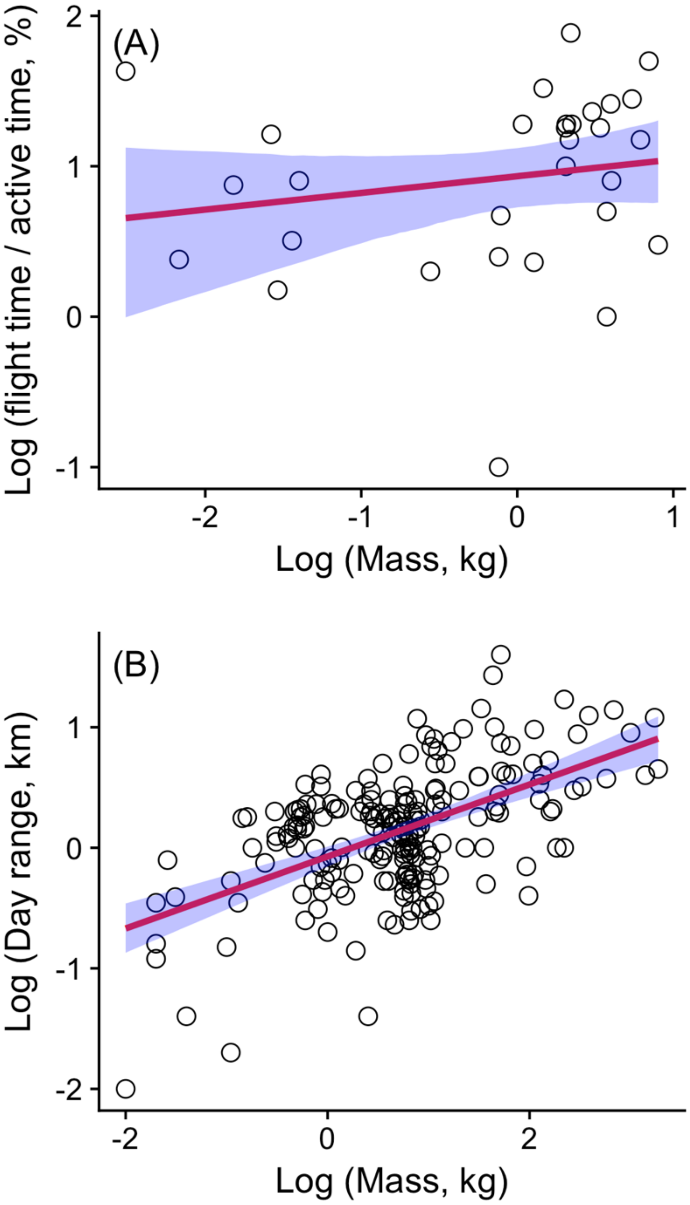

Fig. S4. Fitted ordinary least squares regressions (red lines) for percentage of active time spent flying (*F*) by birds (panel A), and length of day range (*D*) of mammals (panel B) in relation to body mass (*M*). Blue polygons show 95% confidence limits determined by bootstrapping (as described in the main text). For birds, data on flight time (and associated uncertainty) were combined with data on travel speed (and associated uncertainty) (see Fig. S2A) to derive the scaling of day range (assuming an average of 12h of potential activity).

for 143 species of birds and 73 species of terrestrial, non-volant, placental mammals. Fitted parameters for the body mass scaling of FMR (Fig. S5) (with standard errors in brackets) are:

Birds, $\log(FMR) = 3.01\ (\pm\ 0.02) + 0.66\ (\pm\ 0.02) \times \log(M)$

Mammals, $\log(FMR) = 2.89\ (\pm\ 0.03) + 0.73\ (\pm\ 0.02) \times \log(M)$

Using these fitted parameters, we were able to determine the scaling (and associated uncertainty) of the predicted limits to density (see Table 1 in the main text). Here, we also present the scaling of c_M, which indicates the intensity of habitat use under normal conditions of density (see Fig. S6 for scaling under the targeted search model). Derived parameters (with standard deviations across bootstrap replicates in brackets) are:

Systematic search model:

Birds, $\log(c_M) = -0.41\ (\pm\ 0.17) + 1.53\ (\pm\ 0.14) \times \log(M)$

Mammals, $\log(c_M) = -0.96\ (\pm\ 0.08) + 0.81\ (\pm\ 0.06) \times \log(M)$

Targeted search model:

Birds, $\log(c_M) = -2.03\ (\pm\ 0.25) + 1.42\ (\pm\ 0.23) \times \log(M)$

Mammals, $\log(c_M) = -0.88\ (\pm\ 0.11) + 0.51\ (\pm\ 0.08) \times \log(M)$

Here, c_M encompasses the relationship between the scaling of home range area and that of daily travel distance, and reflects the intensity of habitat use. Under the targeted-search model, this is highly divergent for small birds and mammals, but converges for large birds and mid-sized mammals (Fig. S6). Both small birds and mammals, with low values of c_M, use their habitat more intensively than do larger species. However, small birds appear to use their habitat much more intensively than do small mammals. This presumably reflects the scale at which species of different sizes and taxa search for resources, with small birds searching very intensively for resources of relatively low detectability, and larger species in both taxa seeking out more widespread but relatively detectable resources. These findings could have implications for other aspects of ecology, such as the scaling of consumer searching (Pawar *et al.* 2012), or spatial ecology (Jetz *et al.* 2004). At the smallest body sizes, the greater intensity of space use among birds offsets their greater mobility, resulting in predictions of minimum density similar to those for mammals. With increasing body mass, the intensity of space use converges for birds and mammals, and the greater mobility of birds translates into predictions of lower minimum densities.

C. Bootstrapping to determine parameter uncertainty

Various methods exist to estimate prediction uncertainty for quantile regression (Koenker 2015). However, we also wanted to estimate uncertainty in derived properties (such as minimum density and population scope; see main manuscript). Consequently, we used bootstrapping. For example, to account

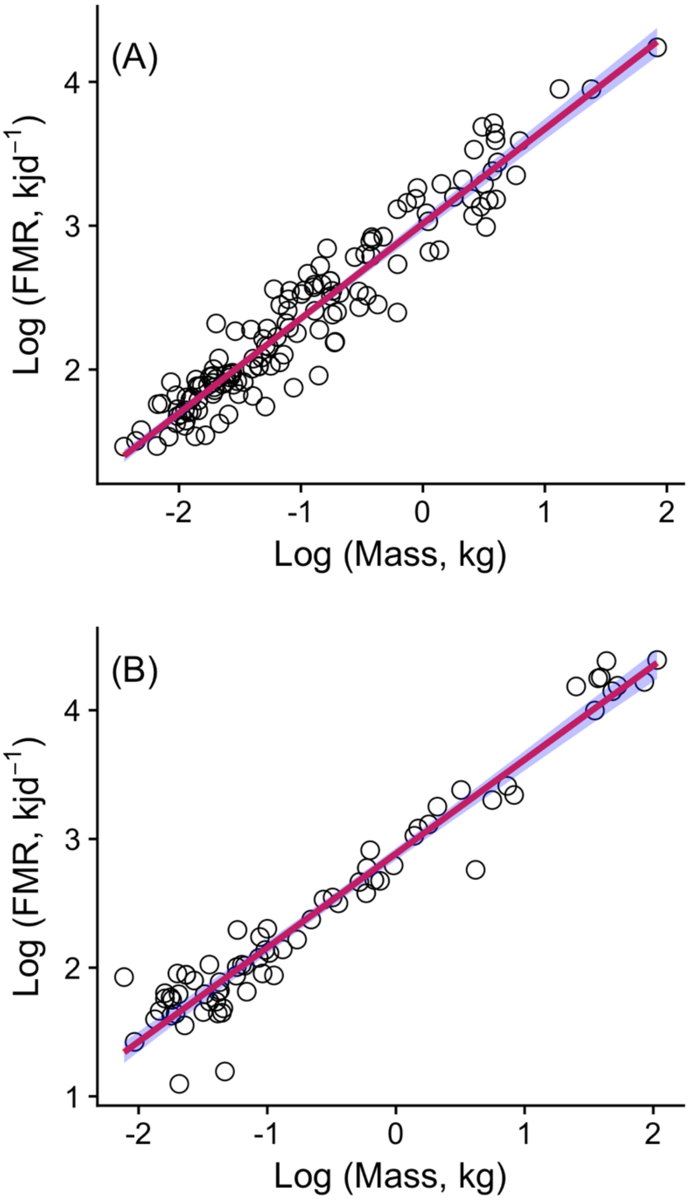

Fig. S5. Fitted ordinary least squares regressions (red lines) for field metabolic rate (Nagy *et al.* 1999; Anderson & Jetz 2005; Speakman & Król 2010) (*FMR*) of birds (panel A) and mammals (panel B) in relation to body mass (*M*). Blue polygons show 95% confidence limits determined by bootstrapping (SI part C). For mammals, we excluded data on non-placentals, marine mammals and volant species.

Table S1. Estimates for bird species of proportion of active day spent in flight

Scientific name	Mass, kg	Percentage of active time spent flying	Source
Sialia mexicana	0.026	16.3	(Mock 1991)
Branta bernicla	1.278	2.3	(Riddington *et al.* 1996)
Auriparus flaviceps	0.007	2.4	(Webster & Weathers 2000)
Himatione sanguinea	0.015	7.5	(Yorinks & Atkinson 2000)
Ardeotis kori	7.994	3.0	(Kemp & Begg 2001)
Bucorvus leadbeateri	3.744	5.0	(Kemp & Begg 2001)
Sagittarius serpentarius	4.017	8.0	(Kemp & Begg 2001)
Circaetus cinereus	2.048	10.0	(Kemp & Begg 2001)
Ephippiorhynchus senegalensis	6.159	15.0	(Kemp & Begg 2001)
Mycteria ibis	2.156	15.0	(Kemp & Begg 2001)
Haliaeetus vocifer	3.400	18.0	(Kemp & Begg 2001)
Necrosyrtes monachus	2.043	18.0	(Kemp & Begg 2001)
Aquila rapax	2.236	19.0	(Kemp & Begg 2001)
Ciconia episcopus	2.061	19.0	(Kemp & Begg 2001)
Anastomus lamelligerus	1.081	19.0	(Kemp & Begg 2001)
Trigonoceps occipitalis	3.016	23.0	(Kemp & Begg 2001)
Polemaetus bellicosus	3.965	26.0	(Kemp & Begg 2001)
Gyps africanus	5.433	28.0	(Kemp & Begg 2001)
Aquila spilogaster	1.466	33.0	(Kemp & Begg 2001)
Torgos tracheliotos	6.969	50.0	(Kemp & Begg 2001)
Terathopius ecaudatus	2.200	77.0	(Kemp & Begg 2001)
Anthochaera phrygia	0.040	8.0	(Oliver 2001)
Falco peregrinus	0.760	2.5	(Palmer *et al.* 2001)
Piranga rubra	0.029	1.5	(Aborn & Moore 2004)
Buteo polyosoma	0.783	4.7	(Baladrón *et al.* 2006)
Lanius senator	0.036	3.2	(Nikolov & Hristova 2007)
Falco peregrinus	0.760	0.1	(Dzialak *et al.* 2009)
Bucorvus leadbeateri	3.744	1.0	(Cooper & Jordan 2013)
Archilochus colubris	0.003	42.9	(Zenzal *et al.* 2014)
Corvus moneduloides	0.277	2.0	(Troscianko & Rutz 2015)

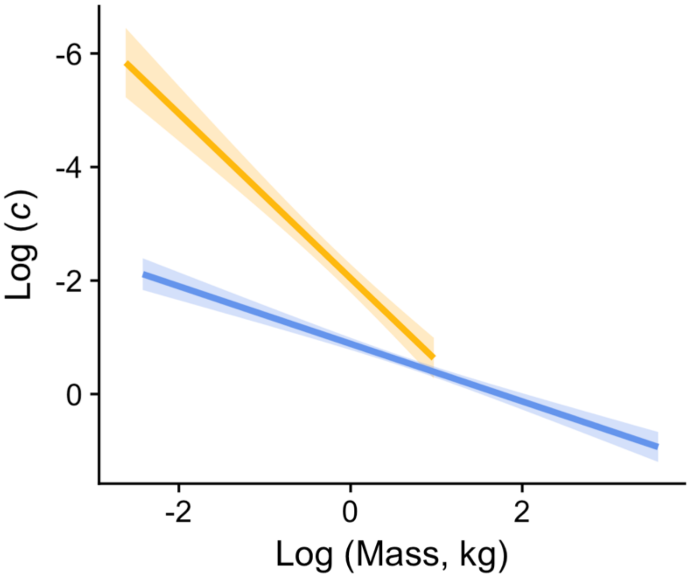

Fig. S6. Derived allometric scaling (solid lines) and associated uncertainty (polygons) in the intensity of space use (c_M) for birds (orange) and mammals (blue), under the targeted-search model. Note that c_M is estimated by dividing home range area by the square of daily travel distance; as such, lower values of c_M indicate a higher intensity of habitat use, which is why the *y*-axis is reversed.

for that uncertainty in evaluating equation 3, which predicts minimum population density on the basis of space use and movement, we drew 2,000 bootstrapped resamples (with replacement) from the relevant data on each of the equation's parameters. To retain structure relative to each original data set, we resampled the raw data from three equally-sized strata with respect to logged body mass. For each bootstrapped sample, we recalculated the scaling parameters for all underlying parameters, and used those to re-evaluate equation 3. For any given logged body mass, confidence intervals were taken as the bounds of the inner 95% of predictions of the lower limit to density, across all 2,000 replicates. The same method was used to determine uncertainty in relationships between empirical density data and body mass, in order to propagate that uncertainty into estimates of population scope.

D. Compiling density, body mass and dietary data for birds and mammals

Taking density data from multiple sources is vulnerable to the possibility of duplicated data points. To reduce this risk, we identified and removed identical estimates for a species in the same study site. For birds, we also removed records associated with colonial and semi-colonial species (densities of which are notoriously difficult to determine (Sanderson *et al.* 2002; Wackernagel *et al.* 2002; Halpern *et al.* 2008)), as well as ratites (Order Struthioniformes), and species with a primary habitat affiliation of wetland, marine or aquatic (based on the IUCN level one habitat classifications obtained from (Birdlife International and NatureServe 2015)). Densities of birds are reported in a range of units. Where these were given in individuals per unit area, the conversion was straightforward. For counts of pairs, nests, territories, singing, calling or courting males, we doubled reported densities.

In addition to density data, we compiled, for each species, estimates of body mass and broad dietary classifications. For birds, body mass data came principally from (Dunning Jr. 2008), supplemented with additional data from (Lislevand *et al.* 2007) and BirdLife International's World Bird Database (http://datazone.birdlife.org/home). Body mass estimates for mammals were largely from the PanTheria data set (Jones *et al.* 2009), or Damuth (1987). For some mammalian species, mass estimates were taken from species-specific studies (often the same sources as those from which density data were obtained). To avoid inconsistencies, we used a single estimate of mass for each species (taking mid-points where mass ranges were given, or averaging across estimates for species for which multiple mass estimates were available). This neglects intra-specific variation in body mass but error in this variable is likely to be very small compared to error in published density estimates.

For birds, dietary data were taken from the same sources as those used to provide density data, and were grouped into the three broad dietary classifications used in our analyses ("carnivore", "omnivore" and "herbivore"). For mammals, the same classifications were obtained initially from the Animal Diversity Web (Myers *et al.* 2016). Species with no classification in ADW were classified using PanTheria (Jones *et al.* 2009) or species-specific studies. A very small number of species were classified based on diets of closely related species.

E. Human population densities

Assessing the scaling of densities and their limits among mammalian guilds provides a context for comparing mammalian population densities with those of human hunter-gatherers, as a result of the shift from more carnivorous diets, to herbivorous and omnivorous diets (Murdock 1967, 1981; Keeley 1988; Burger *et al.* 2017). Data on human hunter-gatherer population densities were obtained from (Keeley 1988; Burger *et al.* 2017). Hunter-gatherers were classified broadly into carnivorous diets based on "simple" hunter-gatherer groups (based on classifications in Keeley (Keeley 1988)) or "fisher/hunter" based on data cited in Burger et al (2017); or omnivorous diets; or largely vegetarian diets (the latter described as "gatherers" in Burger et al). In the case of data from Keeley described as "complex hunter-gatherers", diets were split into omnivores and herbivores by matching the tribal names in Burger et al (2017). Modern human density data were obtained from web sources[1].

Population densities of hunter-gatherer populations were consistent with those of similarly-sized wild mammals (Fig S7A). Indeed, when population scopes were evaluated for 60kg non-human mammals in the three dietary guilds, the density distributions agreed well with human hunter gatherer societies in the corresponding dietary categories (Fig. S7B). However, modern humans live at much higher densities, even when evaluated at a country-wide scale (Fig. S7). At a global scale, the current human density is as high as the maximum for comparably-sized herbivorous non-human mammals; urban populations reach much higher densities, with the highest densities exceeding those of any non-human mammal (Fig. S7A).

[1] Country averages: https://en.wikipedia.org/wiki/List_of_countries_and_territories_by_population_density
Urban densities: https://en.wikipedia.org/wiki/List_of_cities_by_population_density

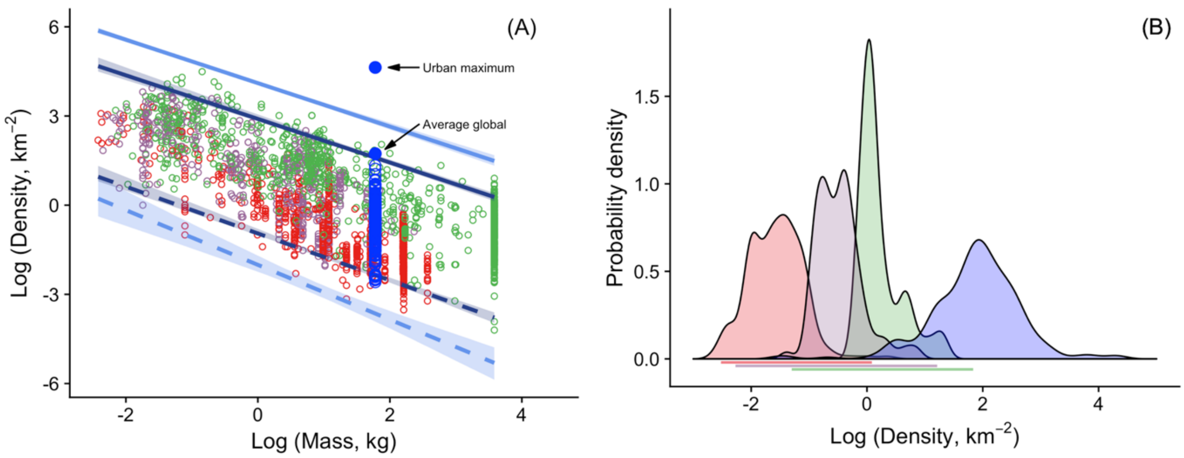

Fig. S7. (A) Collated population density data for mammalian non-human herbivores (green), omnivores (purple), carnivores (red) and human hunter gatherers (open blue circles). Filled blue data points show the global average human population density (total population divided by global land area excluding Antarctica) and the maximum urban density (Manila). Human body mass was assumed to be 60kg. Blue and navy lines show, respectively, the predicted limits (except for those associated with MVP) and the fitted upper and lower quantiles. Broken lines show lower bounds and solid lines show upper bounds. Polygons show 95% confidence intervals associated with predicted and fitted lines. (B) Probability density plots for the population densities of humans. Distributions are shown for hunter gatherers with carnivorous (red) omnivorous (purple) and predominately vegetarian (green) dietary strategies, and for modern humans evaluated at country level (blue). Bars beneath the polygons show the 95% ranges (between upper and lower quantiles) fitted to non-human carnivorous (red), omnivorous (purple) and herbivorous (green) mammals, evaluated for a body mass of 60kg.

References

Aborn, D.A. & Moore, F.R. (2004). Activity Budgets of Summer Tanagers During Spring Migratory Stopover. *Wilson Bull.*, 116, 64–68.

Alerstam, T., Rosén, M., Bäckman, J., Ericson, P.G.P. & Hellgren, O. (2007). Flight speeds among bird species: allometric and phylogenetic effects. *PLoS Biol.*, 5, e197.

Anderson, K.J. & Jetz, W. (2005). The broad-scale ecology of energy expenditure of endotherms. *Ecol. Lett.*, 8, 310–318.

Baladrón, A. V, Bó, M.S. & Malizia, A.I. (2006). Winter Diet and Time-activity Budgets of The Red-backed Hawk (*Buteo polyosoma*) in the Coastal Grasslands of Buenos Aires Province, Argentina. *J. Raptor Res.*, 40, 65–70.

Birdlife International and NatureServe. (2015). *Bird species distribution maps of the world, Version 5.0*. Birdlife International, Cambridge, UK, NatureServe, Alrington, USA.

von Buddenbrock, W. (1934). Über die kinetische und statische Leistung großer und kleiner Tiere und ihre Bedeutung für den Gesamtstoffwechsel. *Naturwissenschaften*, 22, 675–680.

Burger, J.R., Weinberger, V.P. & Marquet, P.A. (2017). Extra-metabolic energy use and the rise in human hyper-density. *Sci. Rep.*, 7, 43869.

Cooper, M. & Jordan, L. (2013). Random time-activity budgets in captive Southern Ground Hornbill *Bucorvus leadbeateri*. *S. Afr. J. Sci.*

Damuth, J. (1987). Interspecific allometry of population density in mammals and other animals: the independence of body mass and population energy-use. *Biol. J. Linn. Soc.*, 31, 193–246.

Dunning Jr., J.B. (2008). *CRC Handbook of Avian Body Masses*. *CRC Handb. avian body masses. Second ed.*

Dzialak, M.R., Carter, K.M., Lacki, M.J., Westneat, D.F. & Andersen, K. (2009). Activity of Post-Fledging Peregrine Falcons in Different Rearing and Habitat Conditions. *Southeast. Nat.*, 8, 93–106.

Halpern, B.S., Walbridge, S., Selkoe, K.A., Kappel, K. V, Micheli, F., D'Agrosa, C., *et al.* (2008). A global map of human impact on marine ecosystems. *Science*, 319, 948–952.

Jetz, W., Carbone, C., Fulford, J. & Brown, J.H. (2004). The scaling of animal space use. *Science*, 306, 266–8.

Jones, K.E., Bielby, J., Cardillo, M., Fritz, S.A., O'Dell, J., Orme, C.D.L., *et al.* (2009). PanTHERIA: a species-level database of life history, ecology, and geography of extant and recently extinct mammals. *Ecology*, 90, 2648–2648.

Keeley, L.H. (1988). Hunter-gatherer economic complexity and "population pressure": A cross-cultural analysis. *J. Anthropol. Archaeol.*, 7, 373–411.

Kemp, A.C. & Begg, K.S. (2001). Comparison of time-activity budgets and population structure for 18 large-bird species in the Kruger National Park, South Africa. *Ostrich*, 72, 179–184.

Koenker, R. (2015). quantreg: Quantile Regression. R package version 5.19. https://CRAN.R-project.org/package=quantreg.

Lislevand, T., Figuerola, J. & Székely, T. (2007). Avian body sizes in relation to fecundity, mating system, display behavior, and resource sharing. *Ecology*.

Marks, E.S. (1948). A Lower Bound for the Expected Travel Among m Random Points. *Ann. Math. Stat.*, 19, 419–422.

Mock, P.J. (1991). Daily Allocation of Time and Energy of Western Bluebirds Feeding Nestlings. *Condor*, 93, 598–611.

Murdock, G.P. (1967). Ethnographic Atlas: A Summary. *Ethnology*, 6, 109–236.

Murdock, G.P. (1981). Atlas of World Cultures. *Murdock 1981 – Atlas World Cult.*, 151.

Myers, P., Espinosa, R., Parr, C.S., Jones, T., Hammond, G.S. & Dewey, T.A. (2016). *The Animal Diversity Web. http://animaldiversity.org*.

Nagy, K.A., Girard, I.A. & Brown, T.K. (1999). Energetics of free-ranging mammals, reptiles, and birds. *Annu. Rev. Nutr.*, 19, 247–77.

Nikolov, B.P. & Hristova, I.P. (2007). Time-Activity Budgets of Juvenile Woodchat Shrikes *Lanius senator* During the Post-Fledging Period. *Ardea*, 95, 235–241.

Oliver, D.L. (2001). Activity budget of the regent honeyeater, *Xanthomyza phrygia*, in northern New South Wales. *Aust. J. Zool.*, 49, 695–712.

Palmer, A.G., Nordmeyer, D.L. & Roby, D.D. (2001). Factors influencing nest attendance and time-activity budgets of peregrine falcons in interior Alaska. *Arctic*, 54, 105–114.

Pawar, S., Dell, A.I. & Savage, V.M. (2012). Dimensionality of consumer search space drives trophic interaction strengths. *Nature*, 486, 485–489.

Riddington, R., Hassall, M., Lane, S.J., Turner, P.A. & Walters, R. (1996). The impact of disturbance on the behaviour and energy budgets of Brent Geese *Branta b. bernicla*. *Bird Study*, 43, 269–279.

Sanderson, E.W., Jaiteh, M., Levy, M.A., Redford, K.H., Wannebo, A. V. & Woolmer, G. (2002). The Human Footprint and the Last of the Wild. *Bioscience*, 52, 891–904.

Speakman, J.R. & Król, E. (2010). Maximal heat dissipation capacity and hyperthermia risk: Neglected key factors in the ecology of endotherms. *J. Anim. Ecol.*, 79, 726–746.

Tamburello, N., Côté, I.M. & Dulvy, N.K. (2015). Energy and the Scaling of Animal Space Use. *Am. Nat.*, 186, 196–211.

Troscianko, J. & Rutz, C. (2015). Activity profiles and hook-tool use of New Caledonian crows recorded by bird-borne video cameras. *Biol. Lett.*, 11, 20150777-.

Wackernagel, M., Schulz, N.B., Deumling, D., Linares, A.C., Jenkins, M., Kapos, V., *et al.* (2002). Tracking the ecological overshoot of the human economy. *Proc. Natl. Acad. Sci. U. S. A.*, 99, 9266–71.

Webster, M.D. & Weathers, W.W. (2000). Seasonal changes in energy and water use by verdins, *Auriparus flaviceps*. *J. Exp. Biol.*, 203, 3333–44.

Yorinks, N. & Atkinson, C.T. (2000). Effects of Malaria on Activity Budgets of Experimentally Infected Juvenile Apapane (*Himatione Sanguinea*). *Auk*, 117, 731.

Zenzal, T.J., Diehl, R.H. & Moore, F.R. (2014). The impact of radio-tags on Ruby-throated Hummingbirds (*Archilochus colubris*). *Condor*, 116, 518–526.
